# Potassii Nitras in the Treatment of Chill and Fever, Clinical Notes

**Published:** 1890-03

**Authors:** J. D. Hunter

**Affiliations:** 352 Tulane Avenue, New Orleans, La.


Dear Sir—Since I placed in your hands for publication my brief paper on *Potassii Nitras*, I have concluded to add my experience in its use for the past few days, which will be suggestive as to the manner of employing the salt.

I was called on February 4th to see a lady from the country, who, for several months, suffered from chill and fever; chill occurred on alternate days. Condition intensely jaundiced, considerably emaciated, entire loss of appetite, exhausted by the least exertion. Ordered thirty grain doses of *Potassii Nitras* to be given two hours before the time of chill and another similar dose when symptoms of chill occurred. No chill took place. The same doses ordered on the next alternate day; no recurrence of chill and no further medication employed. Jaundice cleared up completely, appetite restored, convalescence established. Patient discharged February 12th.

Was called on Wednesday to see a young lady of the city, who was seized with chill and fever the preceding Sunday. Chills occurred at six o'clock every evening. Gave *Quiniæ Sulphas* for two days with no beneficial result. Ordered thirty grain doses of *Potassii Nitras* to be given, as in the case above detailed. No chill took place on the first day; on the second day at the hour of six the usual symptoms of chill were felt. A thirty grain dose of the salt was at once given with the effect of immediate abortion. No further medication. Patient is now attending to her usual household duties.

To-day a young man called at my office, suffering from a small gluteal abscess and a fissure of anus. Upon opening the abscess and cauterizing the fissure he was seized with a violent chill. Administered spirits of ammonia and brandy. Condition unchanged after a lapse of fifteen minutes; temperature 96° S. L. Surface cold and clammy, teeth chattering, face blue and pinched. I thought of *Potassii Nitras* and concluded to hazard a dose; gave thirty grains. Within seven minutes chill ceased, temperature normal, circulation re-established. Within ten minutes from the time of administering the salt the patient was on the street, walking home. This is to me a new experience; heretofore I employed the salt only in chills presumably malarial. To abort a malarial chill has heretofore been difficult—nay, I may say impossible of accomplishment. To abort and at the same time to effect a radical cure—with an approximation towards uniformity, with a few grains of simple salt—has never before been accomplished and is without precedent in medical experience.

Very respectfully yours,

J. D. HUNTER, M. D.

*352 Tulane Ave., New Orleans, La., February 17th, 1890.*

